# Catarrh of the Nose, Throat, and Stomach, of Eight Years’ Standing

**Published:** 1872-11-01

**Authors:** James F. Fitzsimmons


### CATARRH OF THE NOSE, THROAT, AND STOMACH, OF EIGHT YEARS' STANDING.

BY JAMES F. FITZSIMMONS, M. D.

Wm. Bowre, about 47 years old, medium height, bilious temperament, and presenting the appearance of a confirmed dyspeptic, consulted me on the twelfth day of September, 1871, for a chronic difficulty of the stomach, which he said had afflicted him for about eight years. The history of his case, briefly stated, is as follows: He was a soldier during the war of Rebellion, for three years. Exposure caused acute pharyngitis, extending through the posterior fossæ; acute inflammation of the schneiderian membrane was established, and from what I could ascertain, I was led to think that tonsillitis was also present, though whether first, or last, I could not determine. Chronic inflammation, or catarrh of the nose and pharynx, was undoubtedly thus established. The mucous membrane, lining the nasal cavities, pharynx, and very likely extending into œsophagus, in this case, was the seat of an intense inflammation. Whether it is possible for it to have extended down through the œsophagus and attacked the stomach, is a question which I shall not undertake to prove. But the subsequent history leaves us only two theories from which we may decide. Following close upon the pharyngitis, pain, and tenderness of the stomach, with disordered digestion occurred, and continued up to the time when I saw the case. The patient assured me that he had suffered greatly from pain in the stomach, head, and chest, ever since his first attack, and that he was also annoyed by a constant discharge of tough, whitish mucus, from the throat, a thinner and more transparent mucous discharge from the nose, which, during the hours of repose would keep dropping drown from the posterior nares into the throat, from which point it was spat out, or swallowed. When I examined the patient, there was marked tenderness over the epigastrium; percussion over the chest revealed no dulness; ascultation elicited nothing excepting the normal sounds. There was no tenderness over the hypochondria. The bowels appeared undisturbed, though the patient suffered some from constipation. The nose and throat was next examined. The schneiderian membrane was swollen, of a very dark red color. I think it would be better to say that this membrane was hypertropied. The pharynx, posterior part, was streaked from above downward with a stringy, whitish-colored mucus. The mucous membrane was a darker red and in patches, purple color. The tonsils were not enlarged. The patient had been reduced from one hundred and seventy, to one hundred and twenty-five pounds, and he presented a countenance indicative of great suffering; the features were pinched and pointed into a hopeless expression. Diagnosis: catarrh of the nose, throat, and stomach, caused by exposure in the first place, agravated by subsequent colds, unsuitable food, and exhausted by severe military duty (assisted perhaps by chronic diarrhœa).

But why should the stomach continue to be sore and tender after arriving at his home, where he had the best of care; when wholesome food, and good medical attention, both united to aid his restoration to health?

The nutriment could not be digested without intense pain, so much so, that all of the more nutritious food was entirely abandoned, the patient subsisting on small quantities of light, coarse bread, a little milk and tea; and these, he assured me, when partaken of late in the evening, caused so much pain as to keep him awake three-fourths of the night. Eructations were not frequent, nothing but a cutting pain, and burning heat.

Why, again, I asked myself, was it that all the medicine which he had swallowed did him no good? (for he assured me that no permanent benefit had resulted from any medicine he had ever taken) and for years it was his habit to take everything which was prescribed by numerous physicians.

The opinions of physicians differed; some thought his case hopeless, evidently regarding it as malignant; others pronounced it an agravated dyspepsia, and treated it accordingly. After years of suffering and doctoring, he resorted to patent cure-alls, and with how much injury I am not able to say; certain it is with not any benefit.

My conclusion was, as the man was temperate, and prudent, that the secretions from the nose and throat found their way into the stomach, and by reason of their acrid properties, *acted as a continuous irritant.* I informed the patient that this was my opinion, and that it would be necessary to treat the case accordingly, which I proceeded to do, in the following manner: The patient was placed on a chair, with a spittoon directly in front of him. I prepared a solution of nitrate silver, gr. 30 to ounce of water; taking a common catarrhal syringe, I filled it about three-parts full; as much as three drachms of this was thrown up through the posterior nares; the instrument was quickly removed, and the patient instructed to throw his head forward and downward, so as to permit the fluid to pass through, and escape out of the nose; where there is no obstruction this will always occur, provided the patient be always instructed as follows: After being comfortably seated, he should be instructed to open his mouth, fill up the lungs, and hold his breath. The operator must now at once introduce the syringe into the mouth and throat, pass it at once behind the palate, push it upwards as far as the bend (as much as an inch), and throw the contents in as quickly as possible, never giving the patient time to draw his head away. In taking out the syringe, the palate should not be pulled, nor drawn outward by the bulb on the point of the syringe. *Elevate the handle, turn the point downwards at the same time,* and no injury can result. The application in this instance caused a good deal of pain, stermitation and lachrymation, which continued one or two hours. Immediately after the injection, I used a probang, saturated in the same solution, and freely touched every part of the throat, and also passed it for some distance into the œsophagus. Following these applications, there was a great quantity of ropy, whitish mucus, discharged by blowing, and hawking the spittoon was, I should think, half filled with this tough mucus; when poured out, the last of it drew out into a long, stringy mass.

The patient continued to suffer a good deal of pain in his head during the night, and vomited a little he had eaten. The following morning he reported himself as feeling considerably better, had partaken of a little more food than was usual for him, and felt less pain in the stomach. On the second day I used ten grains of nitrate of silver to one ounce of water, and again used the probang, and prescribed the following mixture for the stomach:

| | |
|---|---|
| ℞—Nitro muriat. acid, | ʒ ii. |
| Tinct gentian | ℥ i. |
| Infu. gentian, | ℥ iv. |
| Tinct. card c. | ℥ ss. |

One teaspoonful of which was ordered to be taken before each meal. The medicine was not procured until the following day, but on the third morning he reported himself as feeling decidedly better; had slept soundly and had taken considerably more food, and felt little pain. I now substituted acid carbolic, and glycerine, in place of nitrate silver, and used these with a hand atomizer, showering the throat and nasal cavities, regularly once a day; when there were indications present for the use of nitrate of silver, this was used, after the parts were cleansed thoroughly with the douche and showers of spray.

These measures were persevered in for about six weeks, at the end of which time my patient was entirely well, could eat anything he wanted, and had gained thirty pounds of solid flesh. It is now more than a year, and he has continued to enjoy uninterrupted good health; occasionally yet he has some discharge from his throat, after exposure to cold.

If this case is of any importance, or of any interest to the profession, I will give the treatment of a few cases of nasal catarrh, and ozena.

